# Freedom to read: A personal account of the ‘book famine’

**DOI:** 10.4102/ajod.v3i1.144

**Published:** 2014-11-21

**Authors:** Brian Watermeyer

**Affiliations:** 1 Department of Psychology, Stellenbosch University, South Africa

## Abstract

Even in the digital age, access to literature and other information for people with print impairments remains extremely poor, especially in the developing world. Reading access holds cascading implications for education, economic empowerment, social participation and self-worth. In June 2013 member states of WIPO (the World Intellectual Property Organization) concluded a landmark treaty to reduce copyright impediments to the dissemination of literature to print impaired people. Its effectiveness is not yet clear. Meanwhile, critics hold that disability studies’ analyses have too often lacked insight into the personal and psychological ramifications of exclusion. This article provides an account of the ‘book famine’ from the perspective of a print impaired South African disability researcher, arguing that thorough investigation of the impressions of exclusion is necessary for change. The account highlights the personal, even malignant psychological reverberations of deprivations such as the ‘book famine’, which may carry traumatic effects which cement the status quo.

We read to know that we are not alone. (Nicholson 1990)

## Introduction

As the age of digital information embeds itself, it is tempting to believe that the problem of print access for people with visual and other disabilities is fast becoming a thing of the past. Unfortunately this is not true. Gross inequalities in access to the internet, IT skills training, devices, affordable and accessible literature, as well as the basic educational levels required to engage with the printed word, remain massive obstacles to providing disabled people with the freedom to read. As with so many barriers to participation, technological remedies are available, but not implemented.

For decades international copyright law has been a key stumbling block to literature provision for those who cannot read in the usual way. But in June 2013 member states of WIPO (the World Intellectual Property Organization) finalised a treaty which, it is hoped, will meaningfully improve print access for disabled people. The treaty empowers states to create an exception in national copyright laws, authorising the production of accessible copies of books and other publications for disabled users. The agreement also provides for cross-border sharing of accessible materials between member countries. At present, copyright law dictates that countries produce their own accessible versions, as well as (in most cases) gain individual copyright clearance from publishers. This creates wasteful duplication of capacity amid limited resources, as well as immense inequality between developed and developing nations. Developed countries, such as the United States of America (USA), have large collections of accessible materials which cannot cross borders, leaving the majority of the print impaired population in the world overwhelmingly dependent on local charities and NGOs for literature. For most, this will mean no reading. The World Blind Union estimates that only 7% of published books are ever made accessible in high-income countries; the corresponding figure is less than 1% for poorer nations (WIPO 2014). This appalling circumstance is known as the ‘book famine’. The term is apt.

The USA initially caused consternation by declining to sign the treaty. However, the superpower finally did sign in October 2013, bringing the number of signatory states to 57. We now wait to see which countries will proceed to ratify the treaty, thereby operationalising it in national administrations. In the USA, ratification requires a two thirds majority vote in the Senate, where Republicans have a long history of blocking treaties agreed to by Democrat administrations. There is, therefore, still some way to go. But what does it mean in individual lives to survive without literature?

As someone who has lost reading through a degenerative sight impairment, I followed the lead-up to the WIPO summit in Marrakech with fluctuating feelings. There was a strand of hope, but it was stifled by something else: a dry, cynical and distracted feeling that little would come of it. Later, when the news broke that a treaty had been concluded, the constriction remained. This reaction may seem sensible, as ratification is now the burning question. But I had other reasons. A history of scrounging had filled my relationship to reading with feelings powerful enough to paralyse and undo me.

With this article I try to investigate these feelings, to add some human, personal dimensions to the book famine. I love books, I love reading, and as we shall see, tracing the meaning of inaccess in my life requires exploring many years and layers of self-development. From here the article proceeds as follows: I begin with my own thoughts on (1) what reading can mean in human lives; and (2) why exploring the emotional layers of deprivation is so important for disability studies. After arguing that exclusion always brings not only unequal opportunity, but also an assault on identity, I tell some of my own story of reading. As is the case with auto-ethnography (Ellis & Bochner 2000; Ettorre 2005), I do not claim neutrality or transparency. What I aim to provide is an experiential account showing that disability realities such as the book famine have lasting emotional reverberations which are influential, and deserve attention. The tone of internet postings on the treaty suggests that my thoughts may find some resonance amongst the print impaired community; I await comments with heartfelt interest.

## Reading as freedom

Where food sustains the body, reading can nourish the mind and soul. Reading is an essential conduit to imagining and re-imagining worlds, social and cosmic, material and abstract. Reading literally makes the world bigger, both inside and out, as new possibilities for how to be enrich identity repertoires and deepen personal and social insight. To read is to become more fully a citizen, as democratic processes become better understood, laying a basis for choice. Obviously, reading is how we learn. Not being able to read through poor literacy, poverty or disability is a potential death blow to advancing economic participation. In a very real sense, it is hard to take part in the world without access to the printed word.

Through lifelong development, avenues of reading feed the elaborating of identity. How do I know who I am? What I love? What interests and moves me? I try it on through reading. By reading I taste the world, and develop my tastes for it, growing insight into that which makes my existence meaningful. Without a world of ideas and things to stir my inner sounding board it is harder to know who I am. The alternative is existing in watchful silence, struggling to configure my being in relation to others. To not experience that relatedness is, in a real sense, to not belong. One phenomenological thread of the global disability story is that of an ascribed, inherent difference with the power to undo belonging. Where disablism brings material, emotional or existential homelessness, reading can be a home. Writing can mirror and validate inner experiences eschewed by one’s milieu, shoring up the self as would an attuned human home (Winterson 2011).

But perhaps the most telling truth is the simplest. Reading is a joy. And not being able to read, perhaps especially if one once could, can feel like starvation. To countless print impaired people around the world, I believe it does.

## Deprivation is personal

Elsewhere I have made the case that investigating personal and psychological aspects of disability exclusion is essential to change (Watermeyer 2006, 2009, 2012a, 2012b, 2013), contradicting the dominant, materialist-oriented ‘social model’ view (e.g. Barnes 1998; Barnes, Oliver & Barton 2002; Finkelstein, 1996; Oliver 1990, 2001). Reducing analysis of the book famine to the sheer materiality of services is a miscarriage of our work as social scientists. Deprivation has, perhaps fundamentally, an existential face. If others at all share my experience, the interminable hunger for reading has left deep emotional imprints which can affect identity as well as trust in disability-related support. Surviving grinding deprivation can mean having to suffocate hope. In this position being hopeful feels dangerous, as it echoes down a well of disappointments.

Experiences of unequal resource provision are never subjectively neutral. Instead, it is in our nature to make sense of social contradictions in personal terms. When others are provided for and we are not, somewhere inside the question emerges ‘what is it about me which means I must be left out?’ The question plays with the idea that I am less deserving. Natural defences against the traumatic arbitrariness of unfairness can strengthen the insinuation, as we involuntarily twist and turn to make sense of a contradiction which hurts. Consider the scenario of school children in a Physical Education class being selected by team captains. It is very hard for the last, unchosen child to reflect confidently on the chance inhumanity of the procedure. Despite psychological resistance, he or she is haunted by fantasies about the faults others see. Disabled people are called to take on the inner identity of one who, because of the marker of impairment, is destined to live without that which others are freely given, to not be chosen. Without some psychological co-opting, it is hard to see how global disability inequality could remain as extreme and stubborn as it does (Watermeyer 2012a).

Disability-related deprivation also dovetails with pervasive prejudices about damage and the inability to participate. Together these aspects can take on a dimly held chimera of rationality, based on unseen judgments directed at the self. The slim safety afforded by this position is that it distracts us from the dangerous idea that a different world is achievable. It is safer to be resigned, even reconciled to not having, than to be tormented by a persistent wrong, a contradiction which is always immediate. As an analogy, scholars of poverty explain that it is *relative* deprivation which cuts the deepest. Here, the imbalance between one’s poverty and the wealth of others is understood as injustice, and the pain is excruciating. For colonial peoples interpellated into racist regimes the pain, arguably, is deflected by an attack on the self – I do not have *because of what is wrong with me* (Davids 1996:214). No matter the emotional compromise, entering that part of ourselves in which we feel able to have and deserve will mean confronting conflict and loss.

## Scenes from my story of reading

I began life with full sight, as my eye condition is degenerative. Learning to read was, for me, like falling in love. I read insatiably, filling my young mind with stories, places, creatures, and imaginings. My time of reading freely began around six years old, and continued for a decade. But my sight was degenerating all the while. As I passed the mid-point of my teens, impairment began to intrude on my reading reverie. I found myself straining, letters seemed to jump and disappear on the page, as my fingers involuntarily clutched the book a little tighter. The words were slipping away, and with them my periscope into the world. Losing the words felt like being left behind, left by myself. It is because the body knows water that thirst is so excruciating. Suddenly I had to survive on a little less each day, and then none at all. No books about prehistoric beasts, unsolved mysteries or teenage love affairs. No magazines, no newspapers, no comics. No stories. No food. And somehow no escape. I recall vacillating between frantic disbelief and dull dissociation. The latter was part of an unarticulated, growing sense that loss in my life was somehow predictable, inevitable. A hard inner taskmaster was demanding that I be resigned.

With my mother as reading assistant I achieved a university-entrance certificate at a mainstream school, though performing far below potential. I had been used to easily coming first in my class, and now was not far above average. In my family disability was hardly ever spoken about, whilst at school access provision was by turns absent, grudging, unusable; never something I could trust. As words disappeared off pages, so too did the verbiage of teachers oblivious that their blackboard descriptions, gestures and textbook references were mumbo-jumbo to me. Everyone seemed to be reading, sharing a world of understanding which left me out in the cold. The perception that family members could not bear the reality of my impairment (French 1993; Watermeyer 2009), along with my school’s mix of hostility and neglect, left me apologetic and unentitled as I entered university. My socialisation signalled that admission to the social world required managing my ‘defect’ alone and in silence.

Impostor fears are common amongst university beginners, but mine were acute. What people did here was what I could not do – read. The book famine had set me apart. I remember wandering down a library corridor around the beginning of my first year, with a surreal awareness that I could not extract knowledge from even one of the millions of items that surrounded me. I was supposed to be able to read, and my illegitimacy made me fearful. But much more than that, I *wanted* to read, to feast on all of that knowledge.

Everything at university happened via the printed word. Without conscious reasoning I felt certain that if I ‘confessed’ my inability to lecturers or peers, the bewildered response would be ‘um … so why are you here?’ I sat still in tutorials whilst yet another hefty course reader flopped onto the desk in front of me. I wanted and needed to read but had no way to do so. I moved around the campus, ‘just fill in this form … consult the course handbook … acquaint yourself with the library card abstract system … browse the journal sections’, and so it went on. All the while my stomach lurched with the paradox of a recent, painful loss juxtaposed with the guilt of a trespasser. The tasks all boiled down to one thing: we are here to read books.

I made do, at a university which in the 1980s had no assistive services for sight impaired students. Again my mother stepped into the gap left by disablist society, reading books in anthropology, sociology, literary theory, psychology and much else. At times the stress for both of us was unbearable, as deadlines approached and I was goaded by unread articles piled on my desk. The pain of words so near and yet so far remained barely hidden, appearing as self-blame for my poor organisational skills, my anxiety and procrastination, my laziness. Reading auditorily rather than visually is a skill which must be developed; anxiety makes the process harder. In this modality one is not in control of the words, of the means of production. Inexperienced, I listened too hard, despairing as sentences seemed to fall jumbled in my lap.

Since losing my beloved books at around 16, I had been advised to use South Africa’s postal tape library for print-impaired people, based in Grahamstown. So began a disturbed and addictive relationship. Some people in abusive relationships keep coming back, although never getting what they need. The reasons for this are complex - a mixture of self-defeating compulsion, a sort of desperate hope, and the need to agonizingly re-tell a story of loss from before. Hidden between the lines is the story’s moral: an account of why one did not deserve in the first place. The moral may be so embedded as to make feeling cared for – nourished – virtually impossible.

The library’s selection was tiny, its service neglectful, and its production quality mostly poor. But worst was the waiting. I would request works by a particular author, and spend months watching the postman. Anyone who knows the joy of literature will easily imagine the knife-edge between having and being bereft. To draw a whimsical comparison: imagine the frustration of mislaying a novel one is enthralled by in mid-read. But this novel can never be found. After months of waiting, often nothing would arrive. Or books by authors I would never choose were delivered, mostly thrillers or popular romance novels. Along with these came ‘magazines’ comprising trite, outdated excerpts from the equivalent of village chronicles. On rare occasions a treasure would arrive – a novel by a writer I loved or wanted to explore. Amid the dry waiting, it felt unreal in my hands. Then I might slide in the first cassette to find that it was so poorly read as to be not only unenjoyable, but virtually unintelligible.

When one’s soul has experienced a banquet, it is hard to be thankful for scraps from the kitchen door. ‘This is what you read now’, I felt was being announced to me; ‘this is what blind people read’. I’m sure that my emotional state caused me to throw out several babies with the bathwater, but I couldn’t help it. It was as though the breast had soured. What was offered was just too distant from what I recalled - the blissful freedom of wandering around a library or bookshop, drawing books from the shelves, and sampling what was inside.

It would have helped to approach things in a rational, pragmatic way, by making repeated orders and carefully cultivating relationships with library staff, thereby making the most of the little that was available. But I could not find it in me to do this. Like one trapped in the serial disappointments of an abusive relationship, my actions were driven by conflicted emotion, not reason. A pattern emerged. For long periods I would disengage from both the library and hope, finding a dull security in being clear that I would have no reading. Encouragement from friends would then move me to try again, as a critical voice whispered that being so bereft was mostly my own fault. The real problem, went the self-accusation, was that I was stubborn and ungrateful. Inevitably I again banged my head on disappointment, and re-established my resolve to steer clear of hope. One sees this vacillation in the relationships of people who have suffered traumatic abandonment – in other words, a broken heart. For me, losing the freedom to read had been heartbreaking.

Even now, twenty years later, I carry a self-defeating resistance to proposed solutions to disability-related exclusion. Scepticism is often justified. But below any rational assessment lies a sticky residue of disappointment, deadening creativity and trust in favour of emotional survival. Perhaps this is part of my attachment to persecution, needing to be forgotten and starved to show how forgotten and starved I felt. I think so.

Though never expressing it, I felt irrationally resentful of the reading habits of others. It seemed obscenely wasteful to squander the freedom to read on cheap escapism. Despite my defences, or because of them, I was still drawn to bookshops. Cover titles were legible to me, so I was able to browse. And browse I would, until rising waves of anxiety made me leave, the smell of other people’s books staying in my nostrils.

Many people are baffled by someone who seems sighted but cannot read. When asked to fill in a form in a bank I try to explain as clearly as possible that I cannot see well enough to read. On countless occasions I have been ignored or, more likely, scolded for being irresponsible. ‘You should get glasses!’ is often the irritable response after I have explained for the second or third time. A decade or more after becoming print impaired I still received books on my birthday, even from close friends. Family members continued to direct me to newspaper articles I should read. In a different sort of denial, people expressed to me their gladness at how ‘surely’, there were services ‘out there’ to provide for my needs. It was always hard to disillusion them. To a reader, the notion of ‘no reading’ can stick in the gullet like a fish bone. Many years ago, when I had impressed my non-access to reading on an acquaintance, she exclaimed that without reading she would never be able to be alone. Perhaps she could only be alone with the reassurance reading offered that she was, in fact, not alone.

After postgraduate study I was offered an academic post at a university. With no clear idea how I would manage my responsibilities on the scraps of reading access I had, I accepted. To decline would have felt like giving in to neurotic self-defeat. My familiar impostor feelings began to spiral. ‘If they only knew’, I thought; if my students only knew how little I have read about the theory I am teaching. The stress of not knowing how I would do enough (or any) preparatory reading before a seminar is difficult to explain, with its mix of illegitimacy, shame and something like deceit. The book famine left me feeling useless, like a pretender. At the time, document scanning was becoming available. It was unreliable and slow, but might have been of more benefit to me if I had used it systematically. Instead, it took up the position of an inadequate ‘solution’ which, to my detriment, I unconsciously sidelined.

As student and academic I believed that I had to be more astute than others to perform only well enough, as I would have read a fraction of what my colleagues had. It was imperative that I make maximum use of the little mental food at hand. My assumption, perhaps untrue, was that others were reading voraciously; I had to compensate or disguise my ignorance. I could never, would never, catch up. For a time I tried managing my own small group of volunteer readers, respondents to signs I posted on campus. It didn’t work. Firstly, it felt peculiar to be remunerated for work I could not fulfil without the unpaid assistance of others, especially when I felt I was performing my duties so poorly. Secondly, the precious fragments of reading time were seldom consecutive, providing no continuity as I tried to grasp complex material. Last, readers were often unreliable, and differences in style and ability were difficult to manage. My anxiety was no help. I listened too hard, as my conscience urged me to ‘make the most’ of this minimal resource. Reading with limited assistance is analogous to an infant being fed on schedule rather than demand. It is not need, hunger or readiness that starts the feeding, but external variables which must be accommodated, whether or not the baby is ill, tired, upset or just disinterested. Spontaneity is thwarted, and creativity suffers. After some years of negotiation, my university provided me with a dedicated reading assistant. Part, though not all, of the reason why this took so long was my struggle to believe that my impairment and my ability justified the help. Nothing I recall in my socialisation had indicated that this idea might be true, least of all the book famine itself, and the measly institutional attempts at ameliorating it.

Over perhaps the past eight years the audio-book business, in the form of downloads purchased on the internet, has expanded immensely. This has changed my life. For the first time since the mid-1980s I have an abundance of novels that I can and wish to read. Whilst still limited, the choice in fiction is exponentially larger than before. Still, niches such as my passion for South African writers are not catered for, save for the few local authors with a big international presence. Crucially, non-fiction access remains paltry. In particular, academic books are hardly present at all, since it is an entertainment industry. The target market is primarily ‘mainstream’, comprising nondisabled people who read via audio whilst commuting, working or relaxing. One cannot resist a wry smile at how, when services are aimed at the ‘real world’ of (nondisabled) commercial interest, production standards and availability soar. I have always felt that charity-based service organisations expect something like gratitude from me. By contrast, modern consumers demand immediacy and perfection or will take their currency elsewhere. I see other sight-impaired people feeling at once overjoyed and slighted. But like me, they will get over it. Meanwhile, access to the printed word via scanning and text-to-speech computer software has also improved significantly as the technology has advanced.

The good news above needs heavy qualification. To begin, a commonly held view that scanning technology and computer screen readers provide something like equal access to information is almost pernicious in its falsity. An obvious first point is that this form of access depends wholly on the availability of devices, software and training, far out of reach to most of the developing world. In particular, it is safe to assume that the majority of the world’s sight-impaired population is poor (World Health Organization 2011). Beyond this, scanning is a slow and cumbersome process, rendering documents likely to contain patterns of errors stemming from print quality and font style in the original text. But more significantly, reading via listening to a synthesised computer voice is hard, particularly if the material is challenging. In my experience, a well-produced human-voice recording of a text is overwhelmingly favoured by print-impaired people, but far less available. Further, audio-reading in any form will never allow for the important ability to skim a text, either to gather a few pertinent quotations or ascertain its general usefulness to one’s work. Relying on scanning and text-to-speech, or voice recordings (if available), one commonly has the experience of going to great, time-consuming lengths to bring a book into an accessible form, only to find that it is a worthless irrelevance to one’s interests or research field. If the book has, as most do, only a small number of relevant passages, the audio-reader must listen to every word to happen upon these. For print-impaired people in occupations which require large amounts of reading, such as academics or researchers, live face-to-face reading assistance is thus indispensable. With this assistance, an individual may direct his or her reader in real time to skip some text, focus elsewhere, skim-read, and so on.

Moving to commercial audio-books, access in developing nations such as my own is miniscule, as it also depends on wealth, availability of devices and the internet, and level of education. And these concerns do not even touch upon the question of access to reading material in a mother tongue. South Africa has 10 official languages other than English (11 in total). Non-fiction access, so crucial to education and empowerment, barely exists in the developing world. It is a high-quality, government-funded and large-scale service which is required, and which the WIPO treaty may have brought a little nearer. Access to information is a right, and the book famine continues. It continues to hurt people, in ways which are poorly understood.

## Conclusion

In her memoir *Why be happy when you could be normal?*, Jeannette Winterson (2011) remarks that ‘books don’t make a home – *they are one’* (my emphasis). As a child, Winterson was saved from internalising the hatred of her adoptive mother by encountering alternative worlds in books. In novels she found pictures of herself and her homosexuality different to those engendered by her mother’s paranoia and sadism. In a real sense, books saved her life. Without books the only version of herself imaginable was the one she was fed, a crippling mix of shame and unwantedness. Many millions of disabled people around the world are subject to powerful myths to do with damage, undeserving, shame, and much else. Where there is no home to grow a self capable of creativity, relationship and joy, books can feed the soul. In the words of one commentator, disability has been ‘soaked in shame, dressed in silence, rooted in isolation’ (Clare 1991 cited in Sandahl 2003:44).

These harmful internalisations, coupled with exclusion, make it hard to grow a unified, self-assured and vocal disability minority (Scotch 1988; Watermeyer 2012). In lives where physical access to the world is barred, books are a lifeline to self, an antidote to interpellation. The human need for stories is most acute where systemic symbolic violence foreshortens the flourishing of self. In her life of isolation, every book was for Winterson (2011) ‘a message in a bottle’ sent from a world with different dimensions. ‘The wider we read’, she concludes, ‘the freer we become’. The world’s disabled people need the freedom of reading.
